# Chagas disease control-surveillance in the Americas: the multinational initiatives and the practical impossibility of interrupting vector-borne *Trypanosoma cruzi* transmission

**DOI:** 10.1590/0074-02760210130

**Published:** 2022-07-06

**Authors:** Antonieta Rojas de Arias, Carlota Monroy, Felipe Guhl, Sergio Sosa-Estani, Walter Souza Santos, Fernando Abad-Franch

**Affiliations:** 1 Centro para el Desarrollo de la Investigación Científica, Asunción, Paraguay; 2 Universidad de San Carlos, Laboratorio de Entomología y Parasitología Aplicadas, Ciudad de Guatemala, Guatemala; 3 Universidad de los Andes, Facultad de Ciencias, Centro de Investigaciones en Microbiología y Parasitología Tropical, Bogotá, Colombia; 4 Drugs for Neglected Diseases initiative Latin America, Rio de Janeiro, RJ, Brasil; 5 Centro de Investigaciones en Epidemiología y Salud Pública, Consejo Nacional de Investigaciones Científicas y Técnicas, Buenos Aires, Argentina; 6 Ministério da Saúde, Secretaria de Vigilância em Saúde, Instituto Evandro Chagas, Laboratório de Epidemiologia das Leishmanioses, Ananindeua, PA, Brasil; 7 Universidade de Brasília, Faculdade de Medicina, Núcleo de Medicina Tropical, Brasília, DF, Brasil

**Keywords:** Chagas disease, surveillance, control, interruption of transmission, multinational initiatives

## Abstract

Chagas disease (CD) still imposes a heavy burden on most Latin American countries. Vector-borne and mother-to-child transmission cause several thousand new infections per year, and at least 5 million people carry *Trypanosoma cruzi*. Access to diagnosis and medical care, however, is far from universal. Starting in the 1990s, CD-endemic countries and the Pan American Health Organization-World Health Organization (PAHO-WHO) launched a series of multinational initiatives for CD control-surveillance. An overview of the initiatives’ aims, achievements, and challenges reveals some key common themes that we discuss here in the context of the WHO 2030 goals for CD. Transmission of *T. cruzi via* blood transfusion and organ transplantation is effectively under control. *T. cruzi*, however, is a zoonotic pathogen with 100+ vector species widely spread across the Americas; interrupting vector-borne transmission seems therefore unfeasible. Stronger surveillance systems are, and will continue to be, needed to monitor and control CD. Prevention of vertical transmission demands boosting current efforts to screen pregnant and childbearing-aged women. Finally, integral patient care is a critical unmet need in most countries. The decades-long experience of the initiatives, in sum, hints at the practical impossibility of interrupting vector-borne *T. cruzi* transmission in the Americas. The concept of *disease control* seems to provide a more realistic description of what can in effect be achieved by 2030.

In spite of substantial reductions of prevalence and incidence over the last 3-4 decades, Chagas disease (CD) still imposes a heavy social, economic, and public-health burden on most Latin American countries. Transmission mediated by native triatomine-bug vectors and mother-to-child transmission cause several thousand new infections per year across the continent. Moreover, while an estimate 5-6 million people carry *Trypanosoma cruzi*, access to diagnosis and integral medical care is far from universal.

Starting in the early 1990s, Latin American countries where *T. cruzi* infection is endemic and the Pan American Health Organization-World Health Organization (PAHO-WHO) launched a series of multinational initiatives for the control and surveillance of CD. Here, we present an overview of what those initiatives aimed at, what they have so far achieved, the main challenges they continue to face, and what decades of hard-won experience suggest may be the best ways forward. We follow a north-south course, from Central America-Mexico to the Southern Cone of South America, and close with a summary of a few key, common themes - on the control and interruption of *T. cruzi* transmission, on disease prevention, and on patient care - emerging from this overview. In discussing these common themes, we pay special attention to the CD-specific targets recently set by the WHO in the context of the United Nations 2030 Sustainable Development Goals.^1^

**The Initiative of the Central American Countries and Mexico (IPCAM)**

The Initiative of the Central American Countries for the control of CD (IPCA in its Spanish acronym) was launched in 1997 by Guatemala, Belize, El Salvador, Honduras, Nicaragua, Costa Rica, and Panama; its stated goals were (i) to eliminate the introduced vector, *Rhodnius prolixus*; (ii) to reduce dwelling infestation by native *Triatoma dimidiata*; and (iii) to interrupt blood transfusion-mediated transmission of *T. cruzi*.^2^ The elimination of *R. prolixus*, a highly efficient but non-native (hence entirely domestic) vector, was the top-priority aim.^2^ ^,^ ^3^ ^,^ ^4^ The goal of strengthening CD-specific healthcare within national health systems was incorporated in 2005.^2^ Mexico formally joined the IPCA in 2013, and the acronym of the initiative changed to IPCAM. In line with IPCAM goals, Mexico’s “specific action program” for the control and prevention of CD aims at controlling transmission mediated by house-infesting vectors and eliminating mother-to-child and transfusion-mediated transmission.^5^ Estimates by the WHO (2010) and the Global Burden of Disease Study (GBD; 2019) suggest that ~1.2 to 1.6 million people may be infected with *T. cruzi* in IPCAM countries, and that ~13,000 to 47,000 new infections may be expected to occur annually (Table I).^6^ ^,^ ^7^

**TABLE I:** The burden of Chagas disease in Latin America: 2019 estimates from the Global Burden of Disease (GBD) Collaborative Network.^7^ Estimates include the total numbers of (i) people infected with *Trypanosoma cruzi*, (ii) new (incident) infections, and (iii) disability-adjusted life years (DALYs) lost in 2019. See GBD^7^ and https://www.thelancet.com/gbd for details on the methods used to derive each estimate and its uncertainty interval (UI); the data are available from the GBD Study 2019 at http://ghdx.healthdata.org/gbd-results-tool

Country^*a*^	Initiative^*b*^	People infected by 2019			New infections in 2019^*c*^			DALYs lost in 2019		
		Estimate	UI lower	UI upper	Estimate	UI lower	UI upper	Estimate	UI lower	UI upper
Mexico	IPCAM	1,311,110	1,115,870	1,534,460	36,746	31,350	42,791	12,534	8304	17,462
Guatemala	IPCAM	110,211	92,889	129,231	3975	3354	4692	1252	877	1754
Belize	IPCAM	48	36	65	2	2	3	3	2	6
Honduras	IPCAM	76,313	65,039	88,781	2776	2375	3244	1003	538	1519
El Salvador	IPCAM	35,520	30,013	41,401	1082	911	1259	760	490	1314
Nicaragua	IPCAM	39,908	33,606	46,667	1339	1132	1556	451	317	646
Costa Rica	IPCAM	22,954	18,962	26,926	633	518	744	237	153	341
Panama	IPCAM	21,771	18,234	25,453	636	533	741	248	159	364
Guyana	AMCHA	86	66	112	4	3	5	6	4	12
Suriname	AMCHA	36	26	48	2	1	2	4	3	10
Colombia	IPA/AMCHA	123,430	105,866	144,255	3679	3142	4238	5426	2529	8181
Venezuela	IPA/AMCHA	493,902	429,738	564,220	13,679	12,100	15,402	27,037	18,134	50,565
Ecuador	IPA/AMCHA	132,898	111,732	159,078	3862	3238	4562	1531	1024	2343
Peru	IPA/AMCHA/INCOSUR	217,437	188,881	250,655	6194	5338	7130	2926	2044	4115
Bolivia	AMCHA/INCOSUR	556,181	507,219	611,029	16,786	15,135	18,477	16,882	6156	29,128
Brazil	AMCHA/INCOSUR	2,164,570	1,868,033	2,483,589	63,082	55,296	71,374	174,194	109,040	302,974
Chile	INCOSUR	247,197	219,292	279,688	0^*d*^	-	-	4196	2988	5587
Uruguay	INCOSUR	24,747	21,095	28,855	0^*d*^	-	-	351	238	484
Paraguay	INCOSUR	39,103	33,427	46,034	1244	1066	1444	1541	947	2654
Argentina	INCOSUR	735,491	648,797	838,063	17,207	15,111	19,579	23,553	14,648	61,744
Total		6,352,914	5,508,819	7,298,610	172,928	150,607	197,245	274,135	168,594	491,206

*a*: GBD estimates are not available for French Guiana; *b*: Spanish-name acronyms: IPCAM, Mexico and Central American countries; IPA, Andean countries; AMCHA, Amazonia; INCOSUR, Southern Cone countries; *c*: we note that these estimates are much larger than those published by the World Health Organization (WHO) for 2010 (~39,000 new cases overall);^6^ true values are likely to be intermediate; *d*: assumed to be zero based on certifications of interruption of transmission issued by the Pan American Health Organization (PAHO).

In Guatemala, the Ministry of Health, researchers from San Carlos University, and the Japanese International Cooperation Agency (JICA) outlined the first national control program after several years of baseline research.^8^ Between 2000 and 2005, interventions included area-wide insecticide spraying and measures to prevent transfusion-mediated transmission; the surveillance phase started in 2009.^2^ Drawing on the fruitful Guatemalan experience, JICA supported the establishment of control programs in Honduras and El Salvador. Large-scale insecticide-spraying campaigns began in 2003 and both countries entered the surveillance phase in 2008. By 2009, control and surveillance procedures were also in place across Nicaragua.^2^

Entomological surveillance systems implemented with JICA support strongly rely on community involvement. Homeowners collect suspected vectors in labelled containers and drop them in dedicated “bug mailboxes” set in health posts, schools, or volunteer homes. Staff of the vector control program then visits vector-reporting houses to actively search for bugs and take action as required, including selective insecticide spraying and educational interventions. Vector-control agents engage in active entomological surveillance in villages where there are no “bug mailboxes”.^9^ ^,^ ^10^ ^,^ ^11^

In 2014, JICA discontinued its direct support of vector control activities in Central America. By then, the interruption of *T. cruzi* transmission by *R. prolixus* and a reduction of domestic infestations by *T. dimidiata* had significantly lowered Chagas disease incidence, and coverage of blood-donor screening was 100% throughout the region.^2^ ^,^ ^12^ ^,^ ^13^ ^,^ ^14^ Because JICA-sponsored projects did not include infection diagnosis and treatment, IPCA countries sought alternative support sources; an example of international cooperation towards that end is the “Alliances project” in Guatemala.^15^

The interruption of CD transmission by non-native *R. prolixus* in Mexico and Central America is, together with universal, mandatory blood-donor screening, the most important IPCAM achievement.^2^ By 2011, the PAHO-WHO had certified the elimination of *R. prolixus* from Mexico, Guatemala, Honduras, El Salvador, Nicaragua, and Costa Rica.^2^ ^,^ ^13^ ^,^ ^14^ Along with IPCAM-related activities, insecticide-based malaria-vector control and steadily improving rural housing conditions across the region likely contributed to this elimination.^13^ ^,^ ^14^ Recently, however, *R. prolixus* was found infesting houses and outbuildings in two rural sites of Oaxaca, Mexico - a state that was certified *R. prolixus*-free in 2009 (Table II).^16^ This is a stark reminder of the crucial role of long-term entomological surveillance in first achieving, and then sustaining, the huge progress made by control programs aimed at eliminating non-native vector species.^17^ ^,^ ^18^

The elimination of one non-native species, in any case, leaves a vacant domestic-peridomestic niche that may be taken over by native species.^17^ Decades-long experience from across Latin America shows that insecticide spraying alone cannot eliminate domestic-peridomestic populations of such native triatomines in the long run.^17^ In the IPCAM region, *T. dimidiata* became the main domestic vector in areas once infested by *R. prolixus* (Table II). As a response, integrative control approaches were devised and tested in the region; some of the key components of this research-action program are (i) identifying and reducing dwelling-infestation risk factors; (ii) ascertaining domestic-infestation thresholds below which *T. cruzi* transmission becomes sporadic; and (iii) stimulating gender-sensitive community involvement in, e.g., housing improvement or animal husbandry.^19^ ^,^ ^20^ ^,^ ^21^ ^,^ ^22^ ^,^ ^23^ ^,^ ^24^

**TABLE II:** Main vectors of Chagas disease across the four control-surveillance initiatives in the Americas

Initiative^*a*^	Main triatomine-bug vectors		
	Non-native species^*b*^ (wild populations locally absent; local elimination feasible)	Native species (wild populations widespread; elimination unfeasible)	
		Domestic and/or peridomestic infestation common (and usually starting with dwelling invasion)	Dwelling invasion common (with small, localised, and/or sporadic breeding foci reported in some cases)
IPCAM	*Rhodnius prolixus* ^*c*^	*T. dimidiata* (genotype I and, to a lesser extent, genotype II), *T. barberi*, *T. phyllosoma*, *T. longipennis*, *T. pallidipennis*, *T. picturata*	*T. huehuetenanguensis*, *T. hegneri*, *T. mazzottii*, *T. mexicana*, *T. gerstaeckeri*, *T. nitida*, *T. ryckmani*, *Panstrongylus geniculatus*, *R. pallescens*
IPA	*R. prolixus* ^*d*^ *, Triatoma dimidiata* I^*e*^	*R. prolixus* (Orinoco basin), *T. dimidiata* I (north-western Colombia and possibly northern Venezuela), *R. ecuadoriensis* (I and II), *T. maculata*, *T. carrioni*, *P. chinai*, *P. lignarius/herreri*	*T. nigromaculata*, *T. venosa*, *P. geniculatus*, *P. rufotuberculatus*, *P. howardi*, *R. pallescens*, *R. colombiensis*, *R. robustus* (I)
AMCHA	None recorded so far	*T. maculata*	*R. pictipes*, *R. stali*, *R. brethesi*, *R. barretti*, *R. robustus* (IV and V), *R. montenegrensis*, *R. marabaensis*, *P. geniculatus*
INCOSUR	*T. infestans* ^*f*^	*T. infestans* (south-eastern Bolivia, north-western Paraguay, north-western Argentina)^*g*^ *, T. brasiliensis*, *T. pseudomaculata*, *T. sordida*, *T. rosai*, *T. guasayana*, *P. megistus*, *R. neglectus*, *R. nasutus*	*T. juazeirensis*, *T. lenti*, *T. tibiamaculata*, *T. vitticeps*, *T. costalimai*, *T. rubrovaria*, *T. garciabesi*, *T. eratyrusiformis*, *T. patagonica*, *P. lutzi*, *P. geniculatus*, *P. rufotuberculatus*

*a*: Spanish-name acronyms: IPCAM, Mexico and Central American countries; IPA, Andean countries; AMCHA, Amazonia; INCOSUR, Southern Cone countries; *b*: excluding isolated foci of *Triatoma rubrofasciata*, a tropicopolitan species originally from Asia and tightly associated with rats; *c*: residual foci recently detected in Oaxaca, Mexico; *d*: non-native out of the Orinoco basin (i.e., trans-Andean northern and north-western Colombia); *e*: introduced into coastal Ecuador-Peru; *f*: most likely not native to south-western Peru and parts of northern Chile; further, at least some of the strongly synanthropic populations of the humid Chaco (Paraguay and Argentina) and parts of the dry Chaco (Bolivia, Paraguay and Argentina) probably derive from originally Andean populations that dispersed passively in association with people; a few residual foci persist in southern and north-eastern Brazil; *g*: seemingly wild *T. infestans* found in anthropic landscapes of central Chile may represent feral populations derived from accidentally-introduced, domestic bugs.

As elimination of *R. prolixus* was certified, funding for CD control-surveillance programs declined. Among other effects, this decline limited the capacity of local health services to respond to infestation records arising from community-based surveillance;^11^ insecticide availability and “bug mailbox” maintenance were also affected. Further, vector-control agents working on CD surveillance have historically been redirected to support public-health responses to epidemics and outbreaks of, e.g., dengue and other arboviral diseases; the current Coronavirus disease 2019 (COVID-19) pandemic is obviously having an even larger impact on the operation of local-scale surveillance systems.

*Triatoma dimidiata* and several further native species, including *R. pallescens*, *T. ryckmani*, *T. nitida*, *T. barberi*, *T. pallidipennis*, *T. longipennis*, or the recently described *T. huehuetenanguensis*, are routinely collected inside and around houses across IPCAM countries (Table II). Invasion of houses by (often infected) adult bugs is also common, and some vector-surveillance systems now incorporate a “visitation index” to keep track of this phenomenon; research on the drivers and seasonality of flight-mediated bug dispersal and house invasion has also yielded useful insights.^25^ ^,^ ^26^ ^,^ ^27^ In this scenario of persistent house reinvasion and reinfestation by native vectors, selective insecticide spraying is just one component of a multifaceted, long-term vector control-surveillance strategy that also emphasises gender-sensitive environmental management (particularly at the dwelling level, and covering housing improvements, animal-husbandry practices, waste management, or rodent control) and community involvement.^22^ ^,^ ^23^ ^,^ ^28^ ^,^ ^29^ ^,^ ^30^ ^,^ ^31^ ^,^ ^32^ Overall, it has now become clear that elimination of vector-borne CD is not a feasible goal in any of the countries of the IPCAM initiative - some level of transmission will always exist, and local healthcare systems must be prepared to meet this inescapable challenge.^4^ ^,^ ^22^ ^,^ ^23^ ^,^ ^33^ ^,^ ^34^ ^,^ ^35^

Currently, the Drugs for Neglected Diseases *initiative* (DND*i*), Fundación Mundo Sano, and San Carlos University are working together in Guatemala to include CD diagnosis and patient care in the regular functioning of the Ministry of Health; this will require that health staff at all levels of the system acquire new managerial and clinical skills.^11^ ^,^ ^35^ In sum, although IPCAM-supported action has led to remarkable advances towards reducing the burden of CD in the region, effectively implementing the long-term strategies needed to control CD in Mexico and Central America will require both stronger policies and larger amounts of committed, stable funding.

**The Initiative of the Andean Countries (IPA)**

The Initiative of the Andean Countries for CD control-surveillance (IPA hereafter) was officially launched in 1997 in Bogotá, Colombia within the framework of the Hipólito Unanue Agreement signed by the Ministries of Health of Colombia, Ecuador, Peru, and Venezuela. Its objective was to interrupt vector- and blood transfusion-mediated transmission of *T. cruzi* in the region.^36^ ^,^ ^37^ The IPA was ratified by the Ministers of Health of member countries in November 2002. In the last decade, and to the extent that vector control activities and blood-donor screening were implemented, IPA incorporated new goals focused on (i) providing specific treatment to infected patients; (ii) identifying and treating children infected via mother-to-child transmission; and (iii) the study of acute-disease outbreaks linked to oral *T. cruzi* transmission. Screening of blood donations for *T. cruzi* infection is mandatory in all IPA countries - where, in spite of operational and financial constraints, control interventions against house-infesting vectors have taken place since the launching of the initiative.^38^ ^,^ ^39^ ^,^ ^40^ ^,^ ^41^ ^,^ ^42^ WHO (2010) and GBD (2019) estimates suggest that about 1 million people carry *T. cruzi* in IPA countries, with ~13,000 to 27,000 new infections occurring each year (Table I).^6^ ^,^ ^7^

Three heavily synanthropic triatomine-bug vector species are non-native to IPA countries and have been targeted by area-wide control-surveillance campaigns similar to those successfully deployed against *T. infestans* in Uruguay, Brazil, and parts of Paraguay and Argentina (Table II).^43^ *R. prolixus* is most likely non-native to trans-Andean north-western Colombia (i.e., out of the Orinoco basin) and could hence be locally eliminated. After extensive control efforts across the region, about half of the municipalities considered at high risk of vector-borne CD were certified by the PAHO-WHO as free of transmission mediated by domestic *R. prolixus*.^44^ Similarly, there is compelling evidence that *T. dimidiata* was introduced into western Ecuador-north-western Peru, and insecticide-based vector-control interventions (likely including those against mosquitoes) seem to have had a substantial impact on domestic populations of the species across that sub-region.^43^ ^,^ ^45^ ^,^ ^46^ ^,^ ^47^ ^,^ ^48^ ^,^ ^49^ Finally, introduced *T. infestans* occur in several areas of southern Peru; hence, the country also participates in the Southern Cone Initiative (INCOSUR) and adopts the strategies agreed at INCOSUR meetings for the control of non-native populations of this species (see below and Table II).

Key IPA unmet challenges include timely diagnosis of *T. cruzi* infection and integral patient care. In particular, IPA countries lack dedicated programs for aetiological treatment and clinical follow-up or for the early detection and management of congenital CD. In some disease-endemic regions of Colombia, non-governmental organisations offer CD diagnosis and treatment; to be effective in the long run, however, such worthy efforts must be placed within the context of stronger public healthcare systems.

The progress of vector-control activities has been slow, and interventions are yet to be implemented in some geographical areas where domestic triatomine populations are known to occur. This has been partly due to the lack of adequate knowledge about the ecological and behavioural characteristics of locally native vectors, which generates uncertainty about what control measures and strategies are most appropriate. Native triatomine-bug species are highly diverse (taxonomically, ecologically, and behaviourally) across IPA countries, and vector control-surveillance strategies need to be fine-tuned for species that occur in both domestic-peridomestic and wild habitats (Table II).^39^ ^,^ ^43^ ^,^ ^50^ Findings from Colombia and Venezuela, for example, clearly substantiate the need for surveillance programs capable of gauging the epidemiological risk posed by wild *R. prolixus* populations inhabiting palms of the Orinoco basin.^51^ ^,^ ^52^ ^,^ ^53^ ^,^ ^54^ Recent records of *R. prolixus* in agribusiness plantations of African oil-palms (*Elaeis guineensis*) outline a potentially emerging challenge that is yet to be characterised in terms of human infection risk.^55^ ^,^ ^56^

Wild *R. ecuadoriensis* populations are similarly common in *Phytelephas* palm-crowns across central-western Ecuador.^57^ However, *R. ecuadoriensis* is also a well-known domestic pest in dry inter-Andean valleys of southern Ecuador and northern Peru that lack native palms; since most *Rhodnius* are tightly associated with palms, this suggested the possibility that wild *R. ecuadoriensis* could be absent from the region - and, therefore, that insecticide-based control could eliminate local domestic populations.^58^ However, the discovery that *R. ecuadoriensis* often infest tree-squirrel nests in southern Ecuador,^59^ together with isolated records from Peru,^60^ suggests that wild populations are also present in those dry inter-Andean valleys.

*Triatoma dimidiata* is widely distributed in IPA countries and often colonises in human dwellings, where it can transmit *T. cruzi*; the species has thus become the target of extensive control-surveillance interventions. Wild *T. dimidiata* populations, however, are common in north and central-western Colombia and probably occur also in northern Venezuela.^61^ ^,^ ^62^ ^,^ ^63^ ^,^ ^64^ The species, consequently, is not a candidate for local elimination, and research aimed at developing and testing new, long-term, sustainable control options is - as discussed above for IPCAM countries - critical.

*Panstrongylus lignarius/herreri* is an important domestic vector of *T. cruzi* in the dry middle-upper Marañón valley of north-western Peru.^65^ Other native triatomine-bug species that regularly infest houses and outbuildings in different parts of IPA countries are *T. maculata*, *T. carrioni*, *T. venosa*, *P. howardi*, and *P. chinai*; occasionally, *P. rufotuberculatus*, *T. dispar* or *T. nigromaculata* may also be involved in domestic-peridomestic *T. cruzi* transmission (Table II).^39^ ^,^ ^50^ Although it rarely breeds in human-made structures, *P. geniculatus* has been associated with outbreaks of orally-transmitted, acute CD in Venezuela.^66^ ^,^ ^67^

The scenario outlined above leads to concluding that, in spite of some advances towards preventing and controlling CD in the IPA region, there is still a long way to go. The Andean countries must develop integral and integrated, long-term programs covering all aspects of the disease - from primary prevention to highly-specialised tertiary care. This must include implementing universal antenatal screening of all pregnant women in *T. cruzi*-endemic areas and strengthening national healthcare systems so that they can provide diagnosis, aetiological treatment, and broader care and support to all patients with CD, whether acute or chronic.

**The Initiative of the Amazon Countries (AMCHA)**

Carlos Chagas was the first to report *T. cruzi* from Amazonia.^68^ The parasite was eventually shown to circulate widely among wild mammals and triatomines, but early surveys suggested that human infections were rare and that local triatomine-bug species did not infest houses.^69^ ^,^ ^70^ ^,^ ^71^ ^,^ ^72^ ^,^ ^73^ ^,^ ^74^ ^,^ ^75^ Because endemic transmission of *T. cruzi* was thought to *require* stable house infestation, human infections were interpreted as the result of occasional spillover from sylvatic cycles.^73^ ^,^ ^74^ ^,^ ^75^ ^,^ ^76^ This led to the conclusion that CD was enzootic, but not endemic, in Amazonia, where only sporadic cases occurred in more-or-less discrete geographic clusters.^76^ ^,^ ^77^

This view of Amazonia as “free” from endemic CD was to prevail for decades.^77^ When the 1975-1980 Brazilian national serosurvey reported an average prevalence close to 1% for six Amazonian states,^78^ the results were thought to signal widespread presence of cross-reactive antibodies, immigration of infected people, or labelling or data-processing errors; as Silveira^79^ later put it, “none of those hypotheses […] could be confirmed”. The 2001-2008 Brazilian national survey^80^ did not help clarify the status of the disease in the region. Since the primary goal was to measure the impact of domestic-vector control, sampling was limited to children < 5 years-old; exposure time thus averaged just ~2.5 years, and only six of 14,877 children sampled in Amazonia tested positive.^80^ The survey was hence, in a way, a lost opportunity: wider age-class sampling would have provided a much more faithful picture of regional transmission dynamics, which primarily involve non-domiciliated vectors.^81^ ^,^ ^82^

In the meantime, slowly-accruing evidence started to suggest that CD was more frequent than suspected in Amazonia. New cases were described, several “transmission foci” were identified, infection frequencies > 5% were reported from some sub-regions, and outbreaks of acute CD likely related to food contamination began to crop up at a seemingly increasing rate, mainly in eastern Amazonia.^82^ ^-^ ^96^ In 2002, the European Community-Latin American (ECLAT) network for research on the Triatominae convened a workshop on CD surveillance in Amazonia.^97^ Two years later, the PAHO-WHO launched the Initiative for the Surveillance and Prevention of Chagas disease in Amazonia (AMCHA). Bolivia, Brazil, Colombia, Ecuador, France, Guyana, Peru, Suriname, and Venezuela are members of AMCHA, whose stated goal is to prevent the large-scale establishment of endemic vector-borne CD in the region.^98^

PAHO-WHO-supported AMCHA activities and advocacy, together with popular-media reports on acute-disease outbreaks, started to spread awareness of CD among healthcare workers, the general public, and decision-makers - including those in charge of defining research-funding priorities and disease-notification policies. This set the stage for the generation of new epidemiological, entomological, parasitological, and clinical evidence.^82^ ^,^ ^83^ Below we outline what that evidence, taken as a whole, suggests.

(i) CD is probably (hypo)endemic in Amazonia. Overall prevalence may be about 1-2%, with higher values (~4-5 to > 10%) in some sub-regions (such as the Ecuadorian Amazon or the high-jungle of Peru)^65^ ^,^ ^82^ ^,^ ^84^ ^,^ ^85^ and human groups (such as *Leopoldinia piassaba* palm-fibre gatherers).^77^ ^,^ ^82^ ^,^ ^83^ ^,^ ^87^ For a population of ~34 million, a 1.5% global prevalence would imply that some 500,000 people are infected with *T. cruzi* in Amazonia.

(ii) CD is primarily vector-borne in Amazonia.^82^ ^,^ ^99^ Non-domiciliated native triatomines mediate “classical” transmission through direct human-vector contact and are probably also involved in most food-borne outbreaks; thus, “oral transmission” of CD is essentially vector-borne too.^82^ ^,^ ^83^ ^,^ ^95^

(iii) Classical vector-borne transmission is probably overall more frequent than food-borne transmission. Food-borne cases are just more visible because they tend to be more severe and because active contact-tracing enhances case detection.^82^ ^,^ ^83^ ^,^ ^95^ Underdetection and underreporting, therefore, are in all likelihood much more extensive for the often oligosymptomatic/asymptomatic classical vector-borne infections than for food-borne infections.^81^ ^,^ ^82^

(iv) Outbreaks of food-borne CD cluster heavily in the Brazilian eastern Amazon.^96^ ^,^ ^100^ This is most likely because raw *açaí* (*Euterpe* spp.) juice, often prepared using substandard food-safety practices, is massively consumed in that sub-region. The apparent recent rise of outbreak frequency^100^ parallels local *açaí* production trends: the State of Pará (where > 95% of Brazilian *açaí* is grown) reported a nearly 10-fold rise of *açaí* fruit production between 2000 (150,500 tonnes) and 2018 (1,440,000 tonnes).^101^

(v) Infection with Amazonian *T. cruzi* strains (mainly in TcI, TcIII, and TcIV) can cause severe, even fatal, acute and chronic CD.^77^ ^,^ ^82^ ^,^ ^83^ ^,^ ^91^ ^,^ ^92^ ^,^ ^93^ ^,^ ^102^ ^,^ ^103^

(vi) Wild (and some domestic) mammals make up a huge *T. cruzi* reservoir in Amazonia.^77^ ^,^ ^82^ ^,^ ^103^

(vii) Triatomines are widespread across Amazonia (Table II).^77^ ^,^ ^99^ ^,^ ^104^ ^,^ ^105^ Palms and hollow trees/logs are key ecotopes, but populations of a few species do infest houses.^77^ ^,^ ^99^ ^,^ ^105^ ^,^ ^106^ Wild bugs often invade houses and other premises;^95^ ^,^ ^107^ this behaviour underpins the main mechanism of CD transmission in Amazonia.

Our understanding of the epidemiology of CD in Amazonia has grown substantially, yet much remains to be done. Crucially, most people infected with *T. cruzi* simply remain undiagnosed and therefore do not get the care they need. They deserve better. We now outline some of the most pressing challenges and suggest ways to address them.

(i) We lack reliable estimates of key epidemiological parameters. Prevalence could be estimated through coordinated serosurveys; a complementary/alternative strategy might draw on malaria diagnosis/management networks to collect blood-spot samples and ship them for serological testing. Estimating incidence would ideally require enhanced surveillance/reporting (see below), but data on prevalence by age class can also be used to estimate incidence.^108^ Statistically accounting for imperfect diagnostic-test performance and for underreporting can help improve epidemiological-parameter estimates.^108^ ^,^ ^109^ ^,^ ^110^

(ii) Surveillance is weak and must be strengthened. Notification of acute and chronic CD should be compulsory in all countries. Trained malaria microscopists can help detect *T. cruzi* infections; more generally, primary healthcare workers should be better trained to identify the disease.^111^ Serological screening is done in all blood banks and should be extended to pregnant women.^112^ Death records can also be informative, again with the caveat of underreporting.^113^

(iii) Preventing CD in Amazonia would require a combination of food-safety measures, serological screening of pregnant women, and insecticide-based control of domestic-vector foci. Personal-protection measures for *piaçava*-fibre gatherers, insect-screening of houses and food-processing equipment/premises, and insecticide-impregnated bednets/curtains might help reduce transmission by wild vectors.^114^

(iv) As stressed above, the vast majority of those infected with *T. cruzi* (i.e., tens of thousands) do not receive any specific care in Amazonia. Primary-health workers are overall ill-equipped to identify and manage these patients; key needs include stronger, specific training and a wider availability of diagnostic tests and drugs.^82^

(v) Although food-borne outbreaks have received much attention, awareness of CD is still low in Amazonia. We need to develop better communication/advocacy strategies aimed at health workers, health authorities, researchers, people living at higher risk, and the general public.^115^

This overview suggests that CD is today in Amazonia what CD will likely become across most of Latin America as house infestations are controlled - a hypoendemic disease with some “hotspots” due to more intense exposure to native vectors.^18^ Exposure can be direct (in the wild or when bugs invade or colonise houses) or food-mediated. House-infesting triatomines can be controlled with traditional insecticide-based interventions in the context of long-term surveillance; exposure to wild bugs is less well understood and more research is needed.^18^ ^,^ ^107^ Food-mediated exposure is primarily a matter of food-safety standards; both regulation and communication have important roles to play.^83^ Whatever the origin of the infection (vector-borne, food-borne, or mother-to-child), stronger healthcare and surveillance systems hold the key to reducing the burden of CD in Amazonia.

**The Initiative of the Southern Cone Countries (INCOSUR)**

Created in 1991 by the governments of Argentina, Bolivia, Brazil, Chile, Paraguay, and Uruguay, the Initiative of the Southern Cone Countries (INCOSUR) has played a key role in CD control in the region. Since its inception, INCOSUR has aimed primarily at (i) eliminating house-infesting populations of the main regional vector, *Triatoma infestans*; (ii) reducing/controlling domestic infestations by other (“secondary”) vector species; and (iii) interrupting *T. cruzi* transmission mediated by blood transfusion. INCOSUR provided crucial guidance and drive, both technical and political, to the rest of initiatives.^50^ ^,^ ^116^ Because domestic *T. infestans* populations were widespread over the south-west of the Peru, representatives of this country regularly joined INCOSUR meetings from 1993 on.^50^ Recent estimates suggest that between ~3.5 and ~4.5 million people may carry *T. cruzi* in INCOSUR countries, with perhaps about 100,000 new cases per year (Table I).

Even before INCOSUR was launched, Southern Cone scientists and public-health workers had made fundamental contributions to our understanding of CD aetiology, pathogenesis, management, transmission dynamics, and control.^117^ ^,^ ^118^ Prevention programs focusing on vector control were implemented in Brazil, Argentina, Chile, and Uruguay in the 1960s. By the late 1970s, blood-donor screening was mandatory in all INCOSUR countries.^50^ ^,^ ^119^ Strategies to control mother-to-child transmission began to be developed, tested, and put into practice in the 1980s, mainly in Argentina; because of the perception that vertical transmission of the parasite could not be actually prevented, efforts concentrated on the early diagnosis and treatment of children with congenital infection.^120^

Extensive insecticide-spraying campaigns led to the effective elimination of non-native *T. infestans* populations from Uruguay, Brazil, and parts of Paraguay, Argentina and Peru; the PAHO-WHO eventually certified those areas, as well as two Bolivian departments, as free from *T. cruzi* transmission by that particular vector species.^50^ ^,^ ^121^ As with *R. prolixus* in Mexico,^16^ however, a few *T. infestans* residual foci have since been detected in Brazil, again underscoring the need for long-term surveillance.^122^ ^,^ ^123^ ^,^ ^124^ ^,^ ^125^ Non-native *T. infestans* have also proven difficult to control in urban Arequipa, Peru.^126^ ^,^ ^127^ Importantly, moreover, *T. infestans* is native, and hence widespread in wild environments, across the dry Chaco (Argentina, Paraguay and Bolivia) and the inter-Andean temperate-dry valleys of south-eastern Bolivia.^64^ House reinfestation by native *T. infestans* is common, and transmission of *T. cruzi* can persist or resume (even if at relatively low intensities) in areas under control-surveillance.^128^ ^,^ ^129^ ^,^ ^130^ ^,^ ^131^ ^,^ ^132^ Many other native triatomines, some of which readily infest human dwellings (e.g., *T. brasiliensis*, *T. pseudomaculata*, *T. sordida*, or *P. megistus*), are common in different INCOSUR countries and territories (Table II).^18^ ^,^ ^64^ Hence, vector-mediated transmission of CD should be expected to continue (at relatively low, yet non-zero, rates) in the region - and to disproportionately affect those living in rural substandard houses.^18^ ^,^ ^81^ As noted above, blood-donor screening was also central to INCOSUR goals, and universal coverage was in place by the end of the 1970s.^50^ ^,^ ^116^ ^,^ ^117^ ^,^ ^119^

Control of mother-to-child transmission was incorporated as a third strategic component of national control programs; the strategy is largely based on the serological screening of pregnant women during prenatal care, and on the follow-up of babies born to infected mothers.^133^ ^,^ ^134^ Bolivia, Argentina, Uruguay, and the Brazilian states of Goiás and Mato Grosso do Sul have regulations for universal screening of pregnant women; in Chile, screening covers women living in regions where vector-mediated transmission is considered endemic. Recently, the PAHO-WHO promoted a supranational initiative for the elimination of mother-to-child transmission of CD, HIV, syphilis, and hepatitis B; this “EMTCT Plus” initiative recommends the screening of pregnant women and, when infection is detected, the timely diagnosis, treatment, and follow-up of their children.^135^

Serological surveys, and in particular those involving children living in areas where vector-mediated transmission may still be active, have been used to gauge the impact of control interventions.^80^ ^,^ ^117^ ^,^ ^121^ ^,^ ^136^ Apart from intrinsic methodological difficulties, these surveys have often raised the issue of whether specific anti-*T. cruzi* treatment and adequate follow-up is in effect available to all those testing positive.^18^ ^,^ ^81^ ^,^ ^136^ More generally, offering diagnosis and aetiological treatment to as many people as possible is increasingly seen, together with case notification, as a key component of integrated control programs aimed at reducing the burden of CD and at eliminating it as a public health issue.^1^ ^,^ ^108^ ^,^ ^137^ ^,^ ^138^ While primary-healthcare systems can and should manage most *T. cruzi* infections, in many local settings such systems need to be strengthened with suitably trained staff, appropriate technology, and effective patient referral/counter-referral networks. With adequate training and support, primary-healthcare workers can and should, in addition, actively seek further cases in the families of newly diagnosed patients.^139^ In sum, the current strategy in most INCOSUR countries to reduce the number of people infected with *T. cruzi* combines primary prevention (through vector control-surveillance and blood/organ-donor screening) and secondary prevention (through adequate patient care, including aetiological treatment, and the screening of pregnant women and their offspring).^108^ ^,^ ^138^ ^,^ ^140^

The main challenges faced by INCOSUR countries in their efforts to bring CD under control can be summarised as follows.

(i) Widespread presence of efficient native vectors. This includes native *T. infestans* and several other species that often breed in or around houses and can thus maintain domestic-peridomestic transmission of *T. cruzi* (Table II).

(ii) Insecticide-resistant vector populations. Pyrethroid-resistant *T. infestans* were discovered in Yacuiba, Bolivia, and Salvador Mazza, Argentina, in the 2000s, and later shown to be widespread across the Chaco and in parts of the south-eastern Bolivian Andes.^141^ ^,^ ^142^ ^,^ ^143^ Continuous monitoring of resistance is thus necessary, and further research required to find new alternatives for sustainable vector control.

(iii) Structure of control programs. Decentralisation of the health sector in Latin America since the mid-1980s led to the transfer of most CD control activities to states, provinces, or municipalities.^144^ This brought decision power closer to the communities where interventions are in fact delivered, thus enhancing, to a certain extent, the management of control programs. Decentralisation, however, also had negative effects - on the one hand, it swiftly distributed duties, but, on the other, it overall failed to fairly distribute the resources and expertise needed to tackle those duties, and this generated inequities in the levels of protection enjoyed by different local populations.^144^

(iv) Control of congenital transmission. Mother-to-child transmission is the main mode of *T. cruzi* transmission in vector-free areas within and outside Latin America. The PAHO-WHO estimates that ~15,000 new cases occur each year in Latin America.^134^ ^,^ ^135^ Diagnostic algorithms for congenital CD have well-known limitations, including the low sensitivity of microscopy-based tests, the fact that serological tests cannot be used in newborns because maternal antibodies can result in false-positive results, and the frequent loss to follow-up of initially negative babies.^134^ Molecular tests show much promise, but they need further standardisation and are overall too expensive to be universally accessible.^134^ ^,^ ^145^ There is increasing evidence that congenital transmission can be prevented by treating infected, non-pregnant women of childbearing age with anti-*T. cruzi* drugs.^134^ ^,^ ^146^ ^,^ ^147^ ^,^ ^148^ ^,^ ^149^ ^,^ ^150^

(v) Patient coverage. The large gap between the national demand for specific aetiological treatment and the estimates of CD prevalence and incidence suggests that many patients simply remain undiagnosed, and hence untreated, across INCOSUR countries. Better strategies are critically needed to remove the barriers that keep patients from getting adequate diagnosis and integral care.^151^

(vi) Demonstration of disease burden. Strategic public-health decisions often hinge on the capacity of researchers and program officials to demonstrate disease burden. The visibility of CD, however, is paradigmatically low.^18^ ^,^ ^81^ Compulsory notification of both chronic and acute cases to national surveillance systems could fundamentally help highlight the real burden of the disease.^152^ Notification is now mandatory for all chronic cases in Brazil and for pregnant women and children/adolescents under 18 years of age in Argentina.^153^ ^,^ ^154^

In sum, the long-term INCOSUR experience shows that although *T. cruzi* transmission by non-native vectors, blood transfusion, and organ transplantation can effectively be curbed, preclinical, clinical, social, and implementation research is still needed to achieve the WHO 2030 goals for CD control and prevention.^1^ ^,^ ^108^ ^,^ ^155^ ^,^ ^156^

**In conclusion**

A few common themes emerge from this quick overview of the history, achievements, and challenges of the inter-governmental initiatives for CD control and surveillance. Below we briefly summarise these main topics (see also Table III) and discuss them in the context of the United Nations Sustainable Development Goals and the specific targets set by the WHO to “eliminate Chagas disease as a public health problem” by 2030.^1^ ^,^ ^108^

**TABLE III:** Prospects and hurdles for interrupting *Trypanosoma cruzi* transmission to humans in the Americas

Transmission route		Interruption	Why/how	Status	Loopholes and snags
Transfusion and transplantation		Feasible	Donor-screening regulations	Done	Screening tests not perfect; occasional cases expected
Vertical		Feasible^*^	Screening in routine antenatal care (plus possibly screening and treatment of childbearing-age women in highly-endemic settings)	Not done	Antenatal care not universal; diagnostic tests not perfect; aetiological treatment not 100% effective
Vector-classical	Non-native vectors	Feasible	Chemical control (no wild populations)	Partially done (see Table II)	Surveillance methods not perfect; hidden residual foci; insecticide resistance
	Native vectors	Unfeasible	Wild populations widespread; integral approaches (and stronger, evidence-based advocacy) needed	Some progress, but ultimately undoable	Many native vector species (see Table II); surveillance methods not perfect; underserved human populations; ‘punishment of success’ (low visibility, lack of awareness, low priority); new cases unavoidable
Vector-food	Urban	Feasible	Commercial-food safety regulations	Partially done	Illegal/informal manufacturing/distribution; poor regulation enforcement
	Rural	Feasible	Health promotion, better practices	Not done	Underserved rural populations; occasional cases expected
Accident		Feasible	Field and laboratory biosafety measures		Virtually done	Accidents can always happen; occasional cases expected

*: the primary aim here is secondary prevention of Chagas disease in the child, so ‘interruption of transmission’ is not an accurate description; we note that safety concerns preclude treating pregnant women, but primary prevention of vertical transmission might be achieved by providing aetiological treatment to non-pregnant, childbearing-age women infected with *T. cruzi*.

First, *T. cruzi* transmission mediated by blood transfusion and organ transplantation is effectively under control; because no screening test performs perfectly, however, clinicians should be aware of the possibility that rare, isolated cases arise on occasion. In practice, the WHO 2030 targets of interrupting transmission mediated by blood transfusion and organ transplantation seem both feasible (Table III). Similarly, laboratory or field accidents involving *T. cruzi* (in culture or in its vectors or hosts) can result in sporadic events of transmission (Table III).

Second, the zoonotic nature of *T. cruzi* and the widespread presence of native vectors across the Americas mean that, in practice, interrupting vector-mediated transmission of the parasite is unfeasible (Tables II-III). Stronger, more sensitive surveillance systems with adequate spatial and population coverage are, and will continue to be, critically needed to detect incident cases. Control of non-native vectors was possible because the exclusively domestic-peridomestic habits of introduced bugs rendered them highly vulnerable to insecticide spraying; native vectors, in contrast, persistently reinvade and reinfest insecticide-treated houses and can maintain transmission, even if at slower rates, from the southern United States to Argentina (Tables II-III). Outbreaks of acute CD linked to contamination of food by infected vectors are the most visible, but by no means the only, manifestation of this problem. In general, then, the WHO 2030 target of interrupting transmission mediated by house-infesting vectors, which involves bringing incidence down to zero, seems unfeasible (Table III).

Finally, adequate patient care across all levels of complexity - from primary to tertiary - is a crucial unmet need in most countries. Over the last years, renewed focus on the patient has inspired a good deal of useful discussion about integral care; it seems hardly controversial, however, that most *T. cruzi* infections still remain undiagnosed in Latin America - and that only a fraction of patients with a diagnosis get the care they need. On a more positive note, discussion over patient care has also promoted the view that diagnosing and treating *T. cruzi* infection in women of childbearing age can help prevent mother-to-child transmission. Of course, diagnosing mothers also increases the odds of that, if infected, their babies will be diagnosed and treated. Achieving the WHO 2030 target of interrupting congenital transmission, however, is thought to require ~90% screening coverage of childbearing-aged women, plus treatment of those testing positive and screening of their offspring; there seems to be a long way before these targets are attained in disease-endemic settings (Table III). More generally, providing aetiological treatment to 75% of all people infected with *T. cruzi* - as the WHO targets suggest should be done (together with interruption of transmission by house-infesting vectors, blood transfusion and organ transplantation) to eliminate CD as a public health problem - will require substantial efforts.

By providing sharply defined common goals and encouraging the exchange of expertise and experience between countries, the multinational initiatives coordinated by the PAHO-WHO have played a crucial role in advancing CD control in Latin America. Transmission of *T. cruzi* mediated by blood transfusion and organ transplantation has been interrupted throughout the region, and non-native vectors have been eliminated from most of their past distribution range. Introduced vector populations persist, however, in Peru, Ecuador, Colombia, and parts of Brazil, Chile, or Argentina, and over 100 native species (including all the major domestic vectors) maintain endemic transmission of this zoonotic parasite across the region. Such a scenario suggests that, as continuing control efforts and slowly-improving housing conditions further reduce the incidence of vector-borne infections, prevalence might eventually stabilise at ~1-2% of the population at risk. With ~100 million rural residents in Latin America, it follows that public-health and healthcare systems should be prepared to provide support to a relatively steady pool of at least ~1-2 million *T. cruzi*-infected people. This must include (i) truly universal patient care and antenatal screening and (ii) stronger, continuous, high-coverage control-surveillance systems, both entomological and epidemiological.

The now vast experience of the multinational initiatives and their member countries, in sum, seems to hint at the practical impossibility of interrupting vector-borne *T. cruzi* transmission in the Americas - with “interruption of transmission” defined by the WHO as the “[r]eduction to zero of the incidence of infection caused by a specific pathogen in a defined geographical area, with minimal risk of reintroduction, as a result of deliberate efforts”.^1^ Instead, it would seem that “*disease control*”, which the WHO defines as the “[r]eduction of disease incidence, prevalence, morbidity and/or mortality to a locally acceptable level as a result of deliberate efforts”,^1^ provides a more realistic description of what can be achieved in practice by 2030 and beyond.
